# Lifetime Attributable Risk in Mammography Screenings in Dubai: The Influence of Breast Thickness and Age on Radiation Exposure

**DOI:** 10.3390/diagnostics15010083

**Published:** 2025-01-02

**Authors:** Kaltham Abdulwahid Mohd Noor, Norhashimah Mohd Norsuddin, Iza Nurzawani Che Isa, Muhammad Khalis Abdul Karim

**Affiliations:** 1 Centre of Diagnostic, Therapeutic and Investigative Studies (CODTIS), Faculty of Health Sciences, Universiti Kebangsaan Malaysia, Kuala Lumpur 56000, Malaysia; p103342@siswa.ukm.edu.my (K.A.M.N.); zawaniisa@ukm.edu.my (I.N.C.I.); 2 Dubai Health Academic Corporate, Radiology Department, Rashid Hospital, Dubai 00971, United Arab Emirates; 3 Department of Physics, Faculty of Science, University Putra Malaysia, Serdang 43400, Malaysia; mkhalis@upm.edu.my

**Keywords:** life attribute risk (LAR), mean glandular dose (MGD), organ dose (OD), breast-screening mammography, radiation dose optimization, patient-specific mammography protocols

## Abstract

**Background/Objectives:** This study investigated the lifetime attributable risk (LAR) of radiation-induced breast cancer from mammography screening in Dubai. It aimed to explore the relationship between breast thickness, patient age, and the associated radiation dose during mammographic examinations. **Methods:** A retrospective analysis was conducted on 2601 patients aged 40 to 69 across five screening facilities in Dubai’s healthcare system. Due to a low correlation between the mean glandular dose (MGD) and breast thickness, both glandular and non-glandular doses were included in calculations as the organ dose (OD). This comprehensive approach examined the impact of whole breast tissue on risk assessments. Key exposure parameters such as the kilovoltage peak (kVp), milliampere-seconds (mAs), and source-to-skin distance were extracted from the dose survey. **Results:** Our findings reveal that the organ dose increases with breast thickness, emphasizing the need for dose optimization in denser tissues. The LAR decreases with age across all thickness categories, but higher initial LAR values were seen in younger patients with thicker tissue. This study emphasizes the increased sensitivity of younger women, who usually have denser breast tissue, to radiation-induced cancer risks. **Conclusions:** Personalized screening protocols considering age and breast thickness are crucial for balancing early cancer detection benefits with radiation risks. Future research should improve mammography protocols, explore alternative methods, and consider generic testing for young high-risk patients to mitigate risks while maintaining diagnostic efficacy.

## 1. Introduction

The relationship between mammography screening and the risk of breast cancer is complex and involves factors such as the benefits derived from early detection and detrimental effects from radiation. The mammography density is associated with breast cancer risk and affects breast cancer detection, as it is difficult to see through the dense tissue marking a tumor, as well as to see the tumor against the background of dense tissues. A false-positive can cause patient anxiety, and they end up receiving other unnecessary tests, with possible exposure to radiation, with little or no benefits [1]. Mammography technologies have evolved significantly, with digital mammography now being widely used due to its advantages over traditional film–screen methods. Digital mammography typically delivers lower radiation doses while providing enhanced diagnostic accuracy, particularly in women with dense breast tissue. Studies indicate that digital systems can reduce radiation exposure by approximately 22% compared with analog systems. This reduction is crucial, as cumulative radiation exposure from repeated screenings can increase the risk of radiation-induced breast cancer. Understanding these technological differences is essential for developing personalized screening protocols that optimize both cancer detection and patient safety [2].

However, several concerns are associated with the procedure; for example, the radiation dose, although low in a single examination, accumulates over time and may cause cancer in women, mainly if they are young or have genetic alterations [2,3]. Despite these risks, the positive effects demonstrated by early detection outweigh the negative; this explains its promotion in order to reduce mortality [4,5]. Mammography screening needs to be personalized depending on each woman’s risk and breast tissues and the age at which she commences screening to reduce risk and enhance the benefits [6].

The radiation dose from mammography is expressed using the mean glandular dose (MGD), which is interpreted as the dose that is absorbed by the glandular tissues of the breast. This measure is seen as the most relevant because the glandular tissue is more sensitive to radiation than the fatty tissue is. It is for this reason that various factors affect the MGD, such as the type of mammography equipment used, whether the digital or film–screen method is used, the technical aspects involved in the mammography exam, and the density and size of the breast. In terms of the number of MGDs, a two-view mammogram (craniocaudal [CC] and mediolateral oblique [MLO] views) requires an MGD per view of 1–3 mGy per breast, where digital mammography is often observed to lead to a lower MGD than film–screen mammography [7]. Therefore, the total dose of radiation received through a single screening session would range between 2 and 6 mGy for each breast.

The total amount of radiation exposure from these series of examinations throughout a woman’s life may be predicted depending on the frequency, as well as the years that the screenings are conducted. For example, having yearly mammograms from 40 to 55 years of age combined with biennial mammograms from 56 to 74 years of age leads to the accrual of a significant amount of radiation. For instance, based on reported risks, for a cohort of 100,000 women treated with such a regimen, approximately 86 breast cancer cases and 11 radiation-induced deaths could be expected [3]. Radiation dosimetry for mammography also includes the measurement of the entrance skin dose (ESD), which is the dose received by the surface layer of the skin. Nevertheless, compared with the MGD, the ESD is less effective in providing reliable information on the potential occurrence of cancer. More recent technological developments in mammography include digital breast tomosynthesis, which seeks to facilitate high-quality images with the lowest possible radiation dose and will provide an improved benefit-to-risk ratio for mammographic screening [8].

The rationale for assessing the lifetime attributable risk (LAR) of mammography in the Dubai healthcare system is derived from the observed lack of practice-based investigations about the effects of radiation exposure resulting from mammographic screening. Although international surveys contain comprehensive information regarding the dosages of radiation and probable malignancies, they fail to consider regional factors that can affect risks, including heredity, lifestyle, and healthcare facilities. For instance, it has been estimated that the LAR of fatal breast cancer due to mammography ranges from about 20 to 25 per 100,000 women who undergo screening during the period of 40 to 80 years of age [5]. Nonetheless, these parameters could differ in Dubai because of the disparities in screening techniques, population characteristics, and medical practices. Furthermore, the analysis of specific populations, for instance, young BRCA breast cancer mutation carriers, has revealed that the LAR is significantly higher and may require different screening strategies [9]. Through local studies that focus on Dubai, healthcare providers understand the actual risks associated with the screening of cancer, the right times to conduct the screening, and the right measures to take to enhance the benefits and minimize the negative impacts of radiation on patients.

The concept of LAR is crucial in evaluating the potential risk of radiation-induced breast cancer from mammography [10]. The LAR estimates the probability that an individual will develop cancer due to radiation exposure over their lifetime, factoring in their age at exposure and gender. Studies have shown that digital mammography typically involves an MGD of around 3.7 mGy per view, with annual screenings for women aged 40 to 80 potentially leading to an LAR of 20 to 25 fatal breast cancer cases per 100,000 women [5]. The risk varies significantly with the frequency and starting age of the screenings. For example, Yaffe and Mainprize [3] used an excess absolute risk model to predict that for a cohort of 100,000 women who were screened annually from the ages of 40 to 55 and biennially thereafter until age 74, there would be an estimated 86 induced cancers and 11 deaths due to radiation-induced breast cancer. However, data suggest that while the risk is present, it is relatively small compared with the potential mortality reduction achieved through regular mammographic screening [11,12]. Additionally, a previous study [9] estimated that young BRCA mutation carriers face higher radiation-induced breast cancer risks from mammography, with annual screenings beginning at age 25, resulting in an LAR of 26 per 10,000 women. This highlights the need for tailored screening recommendations based on individual risk factors to balance the benefits and potential harm.

It is thus evident that breast thickness affects the LAR estimation in mammography due to its relationship to the radiation dose. To achieve better clarity, scans taken on thicker breast tissues entail a higher radiation dose, even if the exposure is equal, which raises the radiation risks and chances of cancer. A study revealed that about every extra mm of breast thickness is associated with a considerable rise in the MGD, which is often used to estimate the LAR of radiation-induced breast cancer [13]. First, denser breasts require more cumulated radiation for mammography screening and make tumors less conspicuous, resulting in additional images and, therefore, a higher total radiation exposure [14]. As such, exponentialized factors for screening need to factor in the thickness to enhance the diagnostic value against the risks associated with radiation, especially given the potential variations in the patient population of Middle Eastern healthcare, such as in Dubai, United Arab Emirates (UAE). In the UAE, 21.5% of breast cancer cases in 2014 were diagnosed between the ages of 30 to 40, suggesting a need to consider earlier screening than the currently recommended age of 40 [15,16]. The primary purpose of this study was to assess the lifetime attributable risk (LAR) of radiation-induced breast cancer from mammography screening in women aged 40 to 69 in Dubai. By focusing on the Dubai population, this study sought to fill a crucial gap in our understanding of mammography-related risks in the region, eventually contributing to more personalized and successful breast cancer screening strategies in the region.

## 2. Materials and Methods

The approval for this study was granted by the Dubai Scientific Research Ethics Committee and the Medical Research and Ethics Committee at UKM. This study was conducted retrospectively at five medical facilities in Dubai, with an emphasis on mammography screening. Metadata were collected between January 2019 and July 2022, initially encompassing data from 3000 patients aged 41 to 68. After applying our exclusion criteria, a total of 399 patients were removed from the study. Specifically, [A] patients with breast implants, [B] patients who had undergone mastectomies, and [C] patients with incomplete data were excluded. This resulted in a final sample size of 2601 patients. The metadata included parameters such as age, tube potential (peak kilovoltage, kVp), tube-current–exposure-time product (mAs), compression force (N), breast thickness (mm), X-ray filtration, and anode target. Meanwhile, metadata related to doses were also included and collected, such as the entrance surface dose (dGy) and organ doses (mGy). This information was directly extracted from the DICOM image header. The doses were recorded using the DOSE TQM system (Qaelum NV, Leuven, Belgium) and analyzed in Microsoft Excel. All data views were collected for further analysis. To maintain consistency in the radiation-dose analysis across all patients and to reflect the current practices in Dubai’s breast-cancer-screening program, synthesized 2D images based on the digital breast tomosynthesis (DBT) technique were not included in the evaluation. Thit is because our research specifically examined standard digital mammography data from screening centers to establish baseline lifetime attributable risk (LAR) calculations for the Dubai population. DBT is an advanced imaging technique that provides three-dimensional views of the breast tissue, which can enhance the diagnostic accuracy, particularly in women with dense breasts [17,18]. While DBT is available in the diagnostic center in Dubai, it is not routinely used in screening centers, which are the focus of this study. This focus allows for a more direct comparison of radiation doses and LAR calculations based on the mammography techniques currently employed in Dubai’s screening centers [19].

In this study, the patients’ ages were categorized into three groups: (1) 40 to 49, (2) 50 to 59, and (3) 60 to 69. The age groups were organized based on the women’s breast cancer screening age, which starts from 40 [20]. Meanwhile, the breast thicknesses were categorized into four groups: (1) less than 40 mm, (2) 40 to 59 mm, (3) 60 to 79 mm, and (4) more than 80 mm. This categorization was based on the sampling population to avoid bias [21].

### 2.1. Scanning Acquisition

The five centers involved in this study used the same digital breast mammography unit which was MAMMOMAT Inspiration (Siemens AG, Muenchen, Germany). The X-ray tube has a tungsten (W) or molybdenum (Mo) target, and Mo and rhodium (Rh) filtrations were automatically selected for all imaging techniques. Mammographic images were acquired using the auto-filter mode, where the kVp, mAs, and target/filter combination were chosen automatically based on the compressed breast thickness. The system underwent regular quality control measures to ensure that technical, dosimetry, and image quality standards were met. The images were acquired in the CC and MLO views for each breast.

### 2.2. Dose Measurement

Our preliminary analysis revealed a weak correlation between MGD and breast thickness, indicating that the MGD alone may not adequately represent the radiation exposure experienced by patients with varying breast compositions. Thus, we aimed to focus on the organ dose in this study rather than the commonly used metric, which is the mean glandular dose (MGD). By incorporating both glandular and non-glandular doses into our calculations, we aimed to provide a more inclusive assessment of the total radiation absorbed by the breast tissue. This complete approach improves the accuracy of risk assessments, particularly in populations with diverse breast tissue types. Moreover, using the organ dose allows for a thorough examination of the entire breast tissue’s effect on the radiation risk, which is important for understanding potential biological effects. This focus on the organ dose aligns with recent research suggesting that a broader evaluation of radiation exposure, beyond just the glandular tissue, is vital for accurate cancer risk estimation. By adopting this approach, we were able to confirm that our risk assessments reflect the realities of mammographic screening in our patient population.

A comprehensive approach was employed to accurately calculate the organ dose using the Siemens MAMMOMAT Inspiration mammography system. Initially, patient-specific data were collected, including the compressed breast thickness during the mammogram. Essential exposure parameters such as kVp, mAs, and anode/filter combinations and source-to-skin distance (*SSD*) were extracted from the DICOM metadata of the mammogram. The beam quality, represented by the half-value layer (HVL), is also important to determine the *f* factor.

The entrance surface dose (*ESD*) was calculated using the following equation:

(1) $$ESD=\frac{mAs\times \left(kVp^{2}\right)\times \chi \times BSF}{SSD^{2}}$$

where the output factor, χ, and backscatter factor (BSF) were obtained from the system’s technical documentation. The absorbed organ dose was measured using the following equation:

(2) $$D_{M}=ESD\times f$$

where the *f* factor is obtained from published reference tables based on the breast thickness and HVL. For example, for a breast thickness of 60 mm and an HVL of 0.4 mm Al, the *f* factor is 0.92. The MGD was then calculated by multiplying the *ESD* by the *f* factor, yielding an organ dose of approximately 0.721 mGy.

Regular calibration and validation of the mammography system and dosimetry equipment were performed to ensure accurate dose measurements. All calculated doses and exposure parameters were meticulously documented, and dose reports were generated to ensure compliance with radiation safety standards and guidelines. This methodology ensures precise and reliable calculation of organ doses, contributing to optimal dose calculation.

### 2.3. Radiation Risks

The calculated organ doses were then converted into organ risks using the patient’s age at the time of exposure, as well as gender-specific data obtained from the BEIR VII phase II report through a fitting method. To match the organ risk values to the organ dose, we scaled the organ dose by the actual dose received in relation to a standard dose of 0.1 Gy. Thus, the employed LARs, alongside the organ dose for each of the patients, were converted to organ risks using the following equation:

(3) $$Organ\;risk=\frac{D_{M}}{0.1}\times LAR\;$$

where $D_{M}$ represents the absorbed organ dose for a specific organ calculated in the unit Gy, and *LAR* is the probability per unit dose (Gy) of developing cancer in that specific organ over a lifetime.

Organ risk values were modeled based on the age at exposure, with specific female dose coefficients being given in the BEIR VII phase II report. A third-order polynomial was fitted to the *LAR* coefficient given by the BEIR VII phase II report for various age-at-exposure groups to arrive at the following equation of the *LAR* as a function of the age at exposure, more specifically *Y*. The *LAR* was then determined using the formula in Equation (4) [22]:

(4) $$LAR=b_{0}+b_{1}Y+b_{2}Y^{2}+b_{3}Y^{3}$$

where *b* is the curve fit coefficient, and *Y* is the patient’s age at exposure.

### 2.4. Data Analysis

The statistical analysis was performed using Statistical Packages for the Social Sciences (SPSS) version 25.0. The homogeneity features of the metadata were identified via a one-way ANOVA test. The relationship of the organ dose with the age and breast thickness categories was examined using pairwise comparison tests. To control for potential confounding variables, we stratified our analysis by age groups and breast thickness categories. This stratification allowed us to inspect the effects of these key variables on the organ dose while minimizing the influence of other factors. We calculated descriptive statistics, including means and standard deviations, for various parameters across different age groups and breast thickness categories. This approach highlighted the pattern of trends in the data without making assumptions about complex statistical relationships. The statistical significance level was set at *p* < 0.05 for all analyses.

## 3. Results

### 3.1. Patient Demographic

The demographic data of the patients are summarized in Table 1, which details the mean values for age, breast thickness, and the associated radiation doses. The data are grouped into three age categories (40–49, 50–59, and 60–69 years) to elucidate the variations in organ dose across different ages. Notably, a trend is observed where the organ dose decreases as the age increases, and this effect is further modulated by differences in breast thickness. The findings indicate that younger patients with thicker breast tissue tend to receive higher radiation doses, underscoring the need for tailored screening strategies that account for both age and breast thickness.

### 3.2. Dose–Thickness Correlation

**Figure 1:**
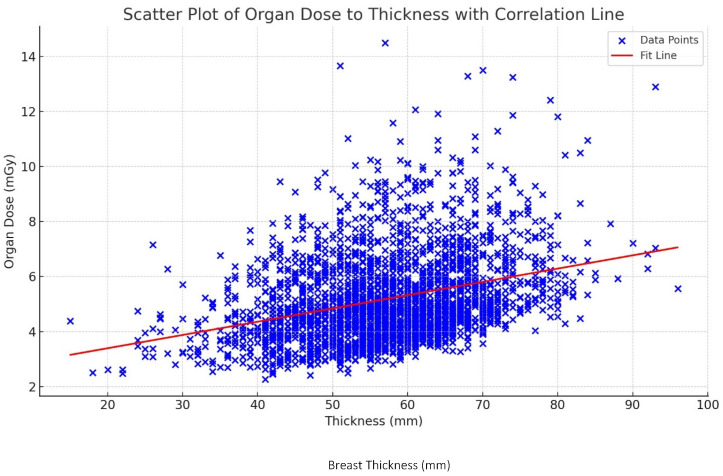
Scatter plot of relationship of dose with breast thickness with correlation line.

The relationship between the organ dose and breast thickness is illustrated in Figure 1. This scatter plot reveals a positive correlation between the breast thickness (mm) and organ dose (mGy), with a correlation coefficient of R = 0.69. As the breast thickness increases, there is a consistent increase in the organ dose, indicating a linear relationship. The data points show a clear upward trend, with organ doses generally rising as the breast thickness increases. This trend is further supported by the fitted correlation line, which rises upward from left to right. The moderate strength of the correlation (R = 0.69) indicates that while breast thickness is an important factor in determining the organ dose, other variables may also influence the dose received. This relationship has important clinical implications. Patients with thicker breast tissue are likely to receive higher organ doses during mammographic examinations. This is because thicker breasts need increased radiation doses to enable proper penetration of the additional tissue and, therefore, maintain the image quality. However, this finding emphasizes the need for careful dose optimization in mammography, particularly for patients with thicker breasts.

### 3.3. Dose Distribution across Age and Thickness

**Figure 2:**
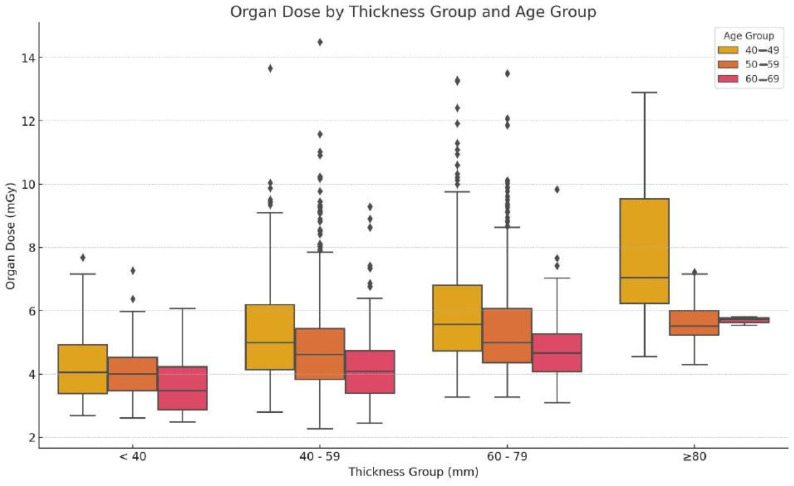
Dose distribution across age and thickness groups.

Figure 2 depicts the organ doses plotted against the thickness and age groups to show the distribution of doses. They are categorized into groups based on breast thickness, i.e., less than 40 mm, 40–59 mm, 60–79 mm, and 80 mm or more. For women aged between 40 and 49, the organ dose rises from below 4 mGy for a thickness of less than 40 mm to almost 8 mGy for a thickness of more than 80 mm. The same trend is noted in the organ doses of the 50–59 and 60–69 age groups, which tend to increase with breast thickness, achieving just under 8 mGy in the category of more than 80 mm thickness. These data reveal that the breast thickness, to a very large extent, affects the organ dose used for assessment in all age groups. The box plot graph illustrates the distribution of organ doses in relation to thickness and age. The organ dose also increases with an increase in thickness, as observed from the increased average median organ dose as the thickness increases across all age groups [23]. In general, for a given thickness, the organ doses are higher in the 40–49 age group compared with the organ doses of the 50–59 and 60–69 age groups.

### 3.4. Statistical Analysis

Table 2 shows the statistical results of the comparison of age group with thickness group. The organ dose exhibits statistically significant variations with the thickness range and age group based on the ANOVA and their p-values. As seen in Figure 2, the organ dose is approximately proportional to thickness, and an increase is observed when the thickness is ≥80 mm. Remarkable variations are observed in the thickness of different age groups within the same classifier, which is particularly the case in the youngest (40–49) and the elder (60–69) age groups. Based on the above findings, it is clear that thickness and age are critical parameters concerning optimization, which means that neither aspect should be overlooked when investigating radiation dose in mammography.

Table 3 presents the imaging parameters across all views. The mean kV settings are slightly higher for the MLO views (LMLO and RMLO) than for the CC views (LCC and RCC). This may imply a preference for slightly higher kV values in oblique views, potentially due to the increased tissue thickness. Both MLO views exhibit higher average mAs settings than the CC views, indicating a higher radiation dose that could be linked to the need to penetrate thicker or denser breast tissue, typically found in oblique views.

The compression force and breast thickness showed variability, likely influenced by the individual patient anatomy and radiographer techniques, with higher values seen in the LMLO and RMLO views for larger or denser breasts. The oblique views show a higher average thickness, which is consistent with the higher kV and mAs settings observed. This supports the notion that oblique views deal with larger volumes of breast tissue, as indicated by the increased parameters needed to achieve sufficient image quality [3,24]. Radiation doses vary across views, with MLO views requiring higher entrance doses and organ doses, particularly in the RMLO view. The entrance doses are higher for the LMLO and RMLO views, and these higher doses correlate with the increased mAs and compression force, potentially compensating for the greater thickness and providing adequate image quality [7]. Monitoring cumulative organ doses, as seen in the LCC view, is crucial for patients undergoing repeated imaging to minimize the risk of radiation-induced effects [14].

**Figure 3:**
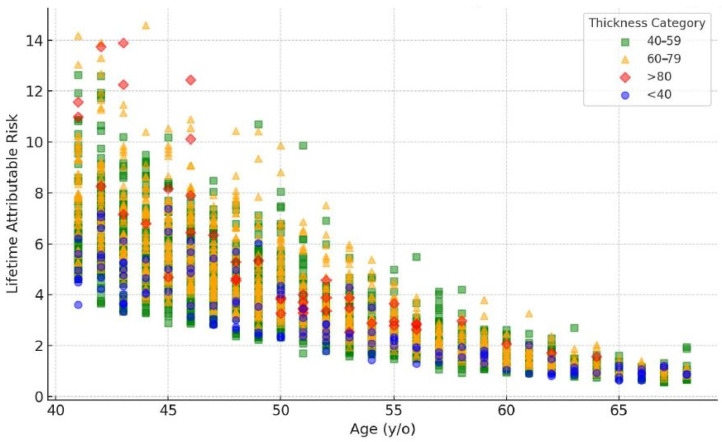
Scatter plot of lifetime attributable risk (LAR) vs. age and breast thickness category.

The graph in Figure 3 displays the LAR of radiation-induced cancer out of 100,000 people with reference to age. The LAR is the highest in the 40–49 age group, followed by the 50–59 age group. However, the LAR for all types of cancer reduces in the 60–69 age group. This trend concurs with the findings that younger women are more likely to receive higher radiation doses due to their increased breast density, which necessitates higher imaging parameters [12]. However, it should be noted that, even in the 60–69 age group, the LAR remains significant, indicating the necessity to continue monitoring women in this age group for mammary cancer and further improving mammography methods to reduce radiation doses and potential risks [9]. Continued optimization of the radiation dose exposure during mammography screening across all ages remains critical [25,26].

**Figure 4:**
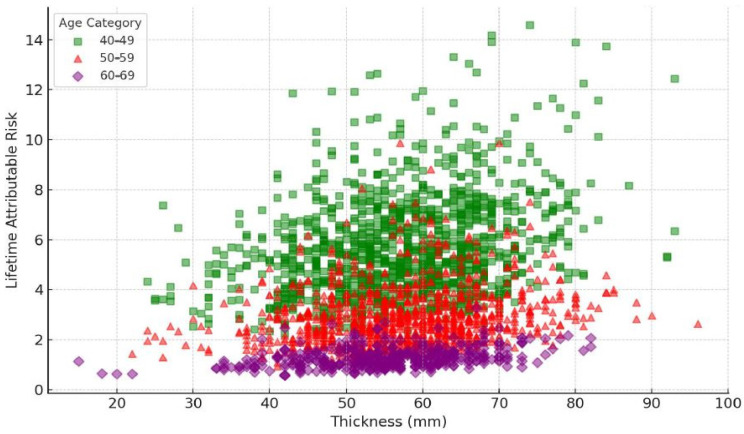
Relationship between breast thickness and LAR across age groups.

Breast thickness has been found to have a significant impact on the amount of radiation absorbed during mammography, leading to higher radiation-dose requirements and potentially increasing the LAR of radiation-induced cancers [27]. This is consistent with the observation that thicker breasts, which have higher densities, are associated with higher initial LAR values. Figure 4 below shows the relationship between breast thickness and LAR.

The scatter plot illustrates a noticeable pattern, where the LAR tends to decrease as the age rises for the same breast thickness. The LAR values in the younger age group (40–49 years) are generally higher than in the older age group (60–69 years) with comparable breast thicknesses. Moreover, an increase in breast thickness is linked to a rise in LAR across all age categories. The data points are clustered more closely within the 40–59 mm thickness range, indicating a prevalent breast thickness range within the sample population.

### 3.5. Summery of Key Results

A strong positive correlation (R = 0.69) was observed between the breast thickness and organ dose. The mean organ dose increased from 4.31 ± 1.17 mGy for breasts < 40 mm thick to 7.93 ± 2.36 mGy for breasts ≥ 80 mm thick in the 40–49 age group. Across all thickness categories, the 40–49 age group consistently received higher organ doses compared with older groups. For instance, in the 60–79 mm thickness category, the mean organ dose was 5.95 ± 1.68 mGy for ages 40–49, compared with 4.78 ± 1.00 mGy for ages 60–69. MLO views required higher average kV (29.8 ± 1.2) and mAs (118.2 ± 45.4) settings compared with CC views (29.3 ± 1.1 kV and 100.7 ± 36.7 mAs), which is likely due to the increased tissue thickness in oblique projections. The lifetime attributable risk (LAR) of radiation-induced cancer per 100,000 people was highest in the 40–49 age group and decreased with age across all breast thickness categories.

## 4. Discussion

### 4.1. Interpretation of Findings

The study findings highlight the critical relationship between breast thickness, age, and radiation dose during mammographic examinations. This is fairly similar to the findings reported for a similar population in Dubai in 2023 by Abdulwahid Noor et al. [28]. The positive correlation between breast thickness and organ dose suggests that patients with thicker breast tissue are exposed to higher radiation doses, which could potentially increase their lifetime attributable risk (LAR) of radiation-induced breast cancer. This observation is consistent with the existing literature, which has demonstrated that breast density and thickness are significant determinants of radiation dose and cancer risk.

It was also observed that the LAR reduced with age in all categories of breast thickness. In one of the studies conducted by Gennaro et al. [29], the authors found that the LAR tended to reduce with age with breast thicknesses below 40 mm, possibly due to the lower tissue density. In the case of the usual LAR in the 40–59 mm tissues, a decrease with age was also noted, but at a greater velocity, which might point to the increased radiosensitivity of tissues due to the higher breast tissue density. This was progressively so in the 60–79 mm category, where the LAR was reduced with the increase in age. From the unadjusted initial LAR, the group with breast thicknesses of more than 80 mm had the highest initial LARs, which decreased rapidly with age. This sharp fall could be understood by the fact that enormous radiosensitivity connected to very dense breast tissue can be observed in young people. Overall, the model provided valuable insights into the relationship between age, breast thickness, and LAR in predicting breast cancer risk. Studies have shown that younger individuals are more sensitive to radiation-induced damage, leading to a higher LAR at a younger age. This is supported by research that uses polynomial models to describe the LAR in relation to age, taking into account changes in population demographics and physiological factors. The observed variability in risk can be attributed to genetic factors that can modulate the radiation risk, suggesting that individual risk assessments could be improved by incorporating genetic profiling.

A significant rise in LAR values is observed in the 40–49 age group across different thickness levels. A considerable number of data points show LAR values surpassing 6, especially in the 40–70 mm thickness range. For individuals aged 50–59, a moderate distribution of LAR values is noted. The majority of LAR values lie between 2 and 8 for the thickness levels of 40–70 mm. In the 60–69 age group, the LAR values are at their lowest. Most LAR values are below 6, even for the higher breast thickness levels.

### 4.2. Comparison with the Literature

Gennaro et al. [29] have developed radiation risk models focusing on the effects of age exposure and breast density in terms of an increased possibility of radiation-induced breast cancer. The models propose that the dependence of the LAR on age is small at high ages, which conforms with the data trends. Da Silva et al. [30] described the role of breast density as mostly being one of masking tumors during a mammography, but also in relation to an inherent risk of cancer and radiation. It is for this reason that this study focuses on the issue of risk assessment for breast cancer and notes that dense breast tissue may be associated with a higher steepness of the first portion of the curve.

The analysis of the LAR across different categories of breast thickness shows a consistent trend: a high initial LAR is found to be related to the thickening of the breast tissue. However, this risk declines with the advance in age. Overall, this suggests that the LAR decreases with age, supporting the hypothesis that younger breast tissue is more sensitive to radiation. This particular discovery is reinforced by current research by Wang [31], who evaluated the effect of the breast density on the efficiency of mammographic screening, as well as the risks involved with radiation. A high breast density was also an issue [24,32,33] in relation to the radiation dose, in that an improvement in the technical parameters requires an increase in the radiation dose in order to obtain high-quality images.

### 4.3. Clinical Implications

The clinical implications of these findings are significant. The increased organ doses observed in patients with thicker breast tissue underscore the need for personalized screening protocols that account for individual patient characteristics [34]. Specifically, dose optimization strategies should be implemented for patients with thicker breasts to reduce their radiation exposure while maintaining diagnostic efficacy. However, it is important to note that excessively lowering the dose could compromise the image quality, leading to ineffective screenings and potentially useless exposure. Therefore, any reduction in dose must be carefully balanced to ensure that the diagnostic competence is not compromised. Additionally, the observed decrease in organ dose with age suggests that mammography protocols might need to be adjusted based on patient age to balance the benefits and risks of screening.

### 4.4. Limitations

While this study provides valuable insights, several limitations should be acknowledged. First, the retrospective nature of the study may introduce selection bias, as the patient population was not randomized. Additionally, this study was conducted in a specific regional context (Dubai), which may limit the generalizability of the findings to other populations. The exclusion of patients with breast implants, mastectomies, or incomplete data may also introduce bias, as these factors could influence the radiation dose and cancer risk. Furthermore, while the statistical analysis was robust, corrections for multiple comparisons were not explicitly mentioned and should be considered to reduce the risk of Type I errors.

### 4.5. Future Directions

Upcoming research should address the above-discussed limitations by conducting prospective studies with more diverse populations to validate our findings and assess their generalizability. Additionally, research exploring alternative screening methods, such as low-dose mammography or other imaging modalities, could provide valuable insights into reducing radiation exposure while maintaining diagnostic accuracy.

On the other hand, exploring the integration of genetic profiling and other risk factors into LAR assessments is a highly recommended future research direction. For example, incorporating the mutation status of the BRCA breast cancer gene or any family history of breast cancer could provide more personalized risk estimates.

## 5. Conclusions

This study provides critical insights into the complex relationship between breast thickness, age, and radiation exposure in mammography screening within Dubai’s healthcare facilities. Our analysis of 2601 patients revealed significant variations in the organ dose and lifetime attributable risk (LAR) across different age groups and breast thickness categories, highlighting the need for a more nuanced approach to breast cancer screening. The strong positive correlation between breast thickness and organ dose underscores the importance of considering individual patient characteristics when developing screening protocols. This finding is mainly relevant for younger patients and those with denser breast tissue, who may be at a higher risk of radiation-induced effects. The observed trends in the LAR across different age groups and breast thickness categories further emphasize the need for tailored screening approaches that balance the benefits of early detection with the potential risks of radiation exposure. These results have important implications for healthcare policy and clinical practice. By providing a comprehensive assessment of radiation risks in the context of Dubai’s population, this study offers a valuable resource for decision-makers seeking to optimize mammography-screening protocols. The data presented here can serve as a foundation for developing risk-stratified guidelines that consider not only age but also breast thickness and other relevant factors.

## Figures and Tables

**Table 1:** Descriptive analysis of acquisition parameters and organ dose.

Category	n	Parameter (Mean ± SD) (Min–Max)						
Age		Age	Tube Voltage (kV)	Tube Current (mAs)	Compression Force (N)	Thickness (mm)	Entrance Dose (mGy)	Organ Dose (mGy)
40–49	1147	45.09 ± 2.55 (41.00–49.00)	29.31 ± 1.1 (26–32)	108.83 ± 39.34 (39–305)	92.89 ± 39.46 (28.4–196.9)	57.3 ± 10.96 (24–93)	4.98 ± 2.05 (1.44–16.02)	5.54 ± 1.66 (2.69–13.66)
50–59	1073	54.00 ± 2.84 (50.00–59.00)	29.36 ± 1.05 (26–32)	98.7 ± 34.52 (33–299)	92.69 ± 38.41 (29.1–194.3)	57.64 ± 10.25 (22–96)	4.57 ± 1.86 (1.15–16.46)	5.09 ± 1.54 (2.27–14.49)
60–69	381	63.16 ± 2.59 (60.00–68.00)	29.13 ± 1.1 (24–32)	81.52 ± 24.66 (34–199)	90.66 ± 38.96 (29.3–190.4)	55.28 ± 10.46 (15–82)	3.78 ± 1.32 (0.98–9.7)	4.38 ± 1.12 (2.45–9.83)
All Patients	2601	51.41 ± 6.91 (41.00–68.00)	29.30 ± 1.08 (24.00–32.00)	100.65 ± 36.70 (33.00–305.00)	92.48 ± 38.95 (28.40–196.90)	57.15 ± 10.62 (15.00–96.00)	4.64 ± 1.92 (0.98–16.46)	5.19 ± 1.59 (2.27–14.49)

**Table 2:** Statistical analysis of organ dose across age groups and breast thickness categories.

Breast Thickness (mm)	Organ Dose (mGy) by Age Group	One-Way ANOVA	Pair-Wise Comparison
40–49 years	50–59 years	60–69 years	40–49 vs. 50–59
<40	4.31 ± 1.17	4.15 ± 0.98	3.64 ± 0.95
40–59	5.25 ± 1.49	4.87 ± 1.51	4.21 ± 1.12
60–79	5.95 ± 1.68	5.44 ± 1.57	4.78 ± 1.00
≥80	7.93 ± 2.36	5.69 ± 0.81	5.69 ± 0.14

**Table 3:** Descriptive analysis of imaging parameters across different mammographic views.

Series	Parameter (Mean ± SD, (Min–Max))					
	Tube Voltage (kV)	Tube Current (mAs)	Compression Force (N)	Thickness (mm)	Entrance Dose (mGy)	Organ Dose (mGy)
LCC	29.3 ± 1.1 (24–32)	100.7 ± 36.7 (33–305)	92.5 ± 38.9 (28.4–196.9)	57.1 ± 10.6 (15–96)	4.63 ± 1.9 (0.98–16.46)	1.21 ± 0.39 (0.56–3.9)
LMLO	29.8 ± 1.2 (24–32)	118.2 ± 45.4 (17–418)	110.6 ± 44.2 (28.2–213.4)	61.9 ± 11.9 (16–100)	5.6 ± 2.5 (1.01–24.2)	1.4 ± 0.5 (0.31–4.66)
RCC	29.7 ± 1.03 (28–32)	109.3 ± 44.2 (50–245)	76.5 ± 20.3 (41.6–119.7)	60.0 ± 11.5 (41–90)	4.9 ± 2.4 (1.83–11.4)	1.24 ± 0.45 (0.65–2.65)
RMLO	29.8 ± 1.17 (24–32)	118.4 ± 46.7 (33–430)	114.4 ± 42.9 (28–198.1)	62.2 ± 11.9 (13–102)	5.7 ± 2.5 (0.89–24.1)	1.38 ± 0.46 (0.57–4.43)

## Data Availability

The data presented in this study are available on request from the corresponding author. The data are not publicly available for ethical purposes.
